# Genomic methods in profiling DNA accessibility and factor localization

**DOI:** 10.1007/s10577-019-09619-9

**Published:** 2019-11-27

**Authors:** David C. Klein, Sarah J. Hainer

**Affiliations:** Department of Biological Sciences, University of Pittsburgh, Pittsburgh, PA 15260 USA

**Keywords:** Chromatin, DNase, MNase, ATAC, ChIP, CUT&RUN, nucleosome occupancy, transcription factors, genomics

## Abstract

Recent advancements in next-generation sequencing technologies and accompanying reductions in cost have led to an explosion of techniques to examine DNA accessibility and protein localization on chromatin genome-wide. Generally, accessible regions of chromatin are permissive for factor binding and are therefore hotspots for regulation of gene expression; conversely, genomic regions that are highly occupied by histone proteins are not permissive for factor binding and are less likely to be active regulatory regions. Identifying regions of differential accessibility can be useful to uncover putative gene regulatory regions, such as enhancers, promoters, and insulators. In addition, DNA-binding proteins, such as transcription factors that preferentially bind certain DNA sequences and histone proteins that form the core of the nucleosome, play essential roles in all DNA-templated processes. Determining the genomic localization of chromatin-bound proteins is therefore essential in determining functional roles, sequence motifs important for factor binding, and regulatory networks controlling gene expression. In this review, we discuss techniques for determining DNA accessibility and nucleosome positioning (DNase-seq, FAIRE-seq, MNase-seq, and ATAC-seq) and techniques for detecting and functionally characterizing chromatin-bound proteins (ChIP-seq, DamID, and CUT&RUN). These methods have been optimized to varying degrees of resolution, specificity, and ease of use. Here, we outline some advantages and disadvantages of these techniques, their general protocols, and a brief discussion of their development. Together, these complimentary approaches have provided an unparalleled view of chromatin architecture and functional gene regulation.

## Background

All DNA-templated processes that occur in eukaryotic cells do so in the context of chromatin. Chromatin is composed of an array of nucleosomes consisting of 147 base pairs of double-stranded DNA wrapped around an octamer of histone proteins (Kornberg and Lorch 1999). Chromatin is highly regulated to facilitate proper function of DNA-templated processes at the levels of individual nucleosomes, DNA accessibility, and higher-order structures—all of which are regulated by chromatin-interacting factors. These chromatin-interacting factors are directed to regions of the genome as both a cause and consequence of local chromatin architecture, creating discrete patterns of factor localization. What emerges is a complex system of reciprocity in which chromatin regulatory factors affect nucleosome architecture, which in turn affects the binding of new regulatory factors. With the dynamic interplay between these processes, diverse methods are necessary to examine nucleosome architecture and regulatory factor binding.

**Fig. 1:**
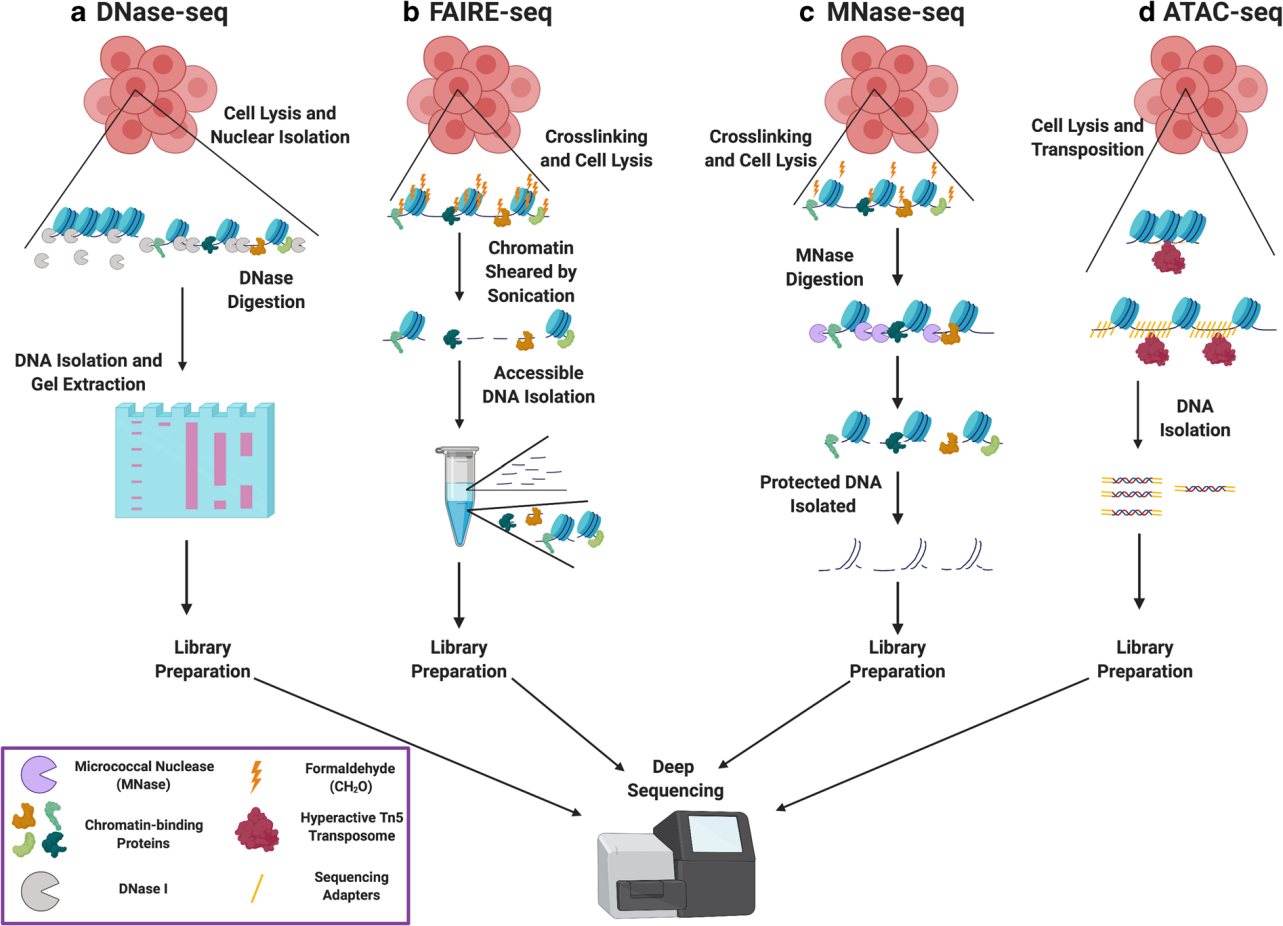
Methods for mapping genome accessibility. **A** DNase-seq identifies open regions of chromatin. DNase-seq relies upon preferential digestion of regions of chromatin that are unprotected by bound proteins, leaving behind accessible regions that are known as DNase I hypersensitive sites (DHSs). **B** FAIRE-seq is dependent on crosslinking of chromatin-interacting proteins to DNA using formaldehyde. Chromatin is then sheared, and regions that are unbound by proteins (e.g., histones) remain in the aqueous layer of a phenol-chloroform extraction, while crosslinked DNA remains in the organic layer. **C** MNase-seq profiles nucleosome occupancy and positioning. After formaldehyde crosslinking, added MNase digests DNA that is unprotected by bound proteins, allowing one to infer increased accessibility by decreased presence in sequencing library. **D.** ATAC-seq relies on the hyperactive Tn5 transposase to insert sequencing adapters at accessible regions of the genome. Following transposition, genomic DNA can be isolated and amplified by PCR, then subjected to deep sequencing. Figure created with Biorender.com

Regulatory elements within a cell are primarily found at open or accessible regions of the genome. Identifying cell-specific regulatory elements is therefore primarily accomplished through accessibility assays. Detecting open chromatin can also identify binding sites for chromatin-interacting proteins. In this review, we will first discuss techniques in the field of chromatin biology for examining chromatin accessibility—including digestion with DNase I and deep sequencing (DNase-seq) (Crawford et al. 2006a, b; Sabo et al. 2006; Song and Crawford 2010), formaldehyde-assisted isolation of regulatory elements (FAIRE-seq) (Giresi et al. 2007; Simon et al. 2012), micrococcal nuclease (MNase) digestion followed by deep sequencing (MNase-seq; (Cui and Zhao 2012a; Henikoff et al. 2011; Mieczkowski et al. 2016; Ramani et al. 2019), and an assay for transposase accessibility (ATAC-seq; (Buenrostro et al. 2013, 2015; Chen et al. 2016; Corces et al. 2017); Fig. 1). These techniques provide important context for gene regulation, especially with respect to nucleosome occupancy and positioning.

Importantly, the genomic location of factors or histone proteins cannot be predicted in cell types by DNA sequence or accessibility alone. Individual protein profiling technologies are therefore used to identify the cell-specific characteristics of functional binding. We will discuss techniques for determining factor binding to and localization on chromatin, including chromatin immunoprecipitation (ChIP) (Albert et al. 2007; Furey 2012; Gilmour and Lis 1984; Gilmour et al. 1991; O’Neill 2003; Solomon and Varshavsky 1985), DNA adenine methyltransferase identification (DamID; (Greil et al. 2006; van Steensel and Henikoff 2000), and chromatin immunocleavage-derived techniques (ChIC/CUT&RUN; (Schmid et al. 2004; Skene and Henikoff 2017) Fig. 2).

**Fig. 2:**
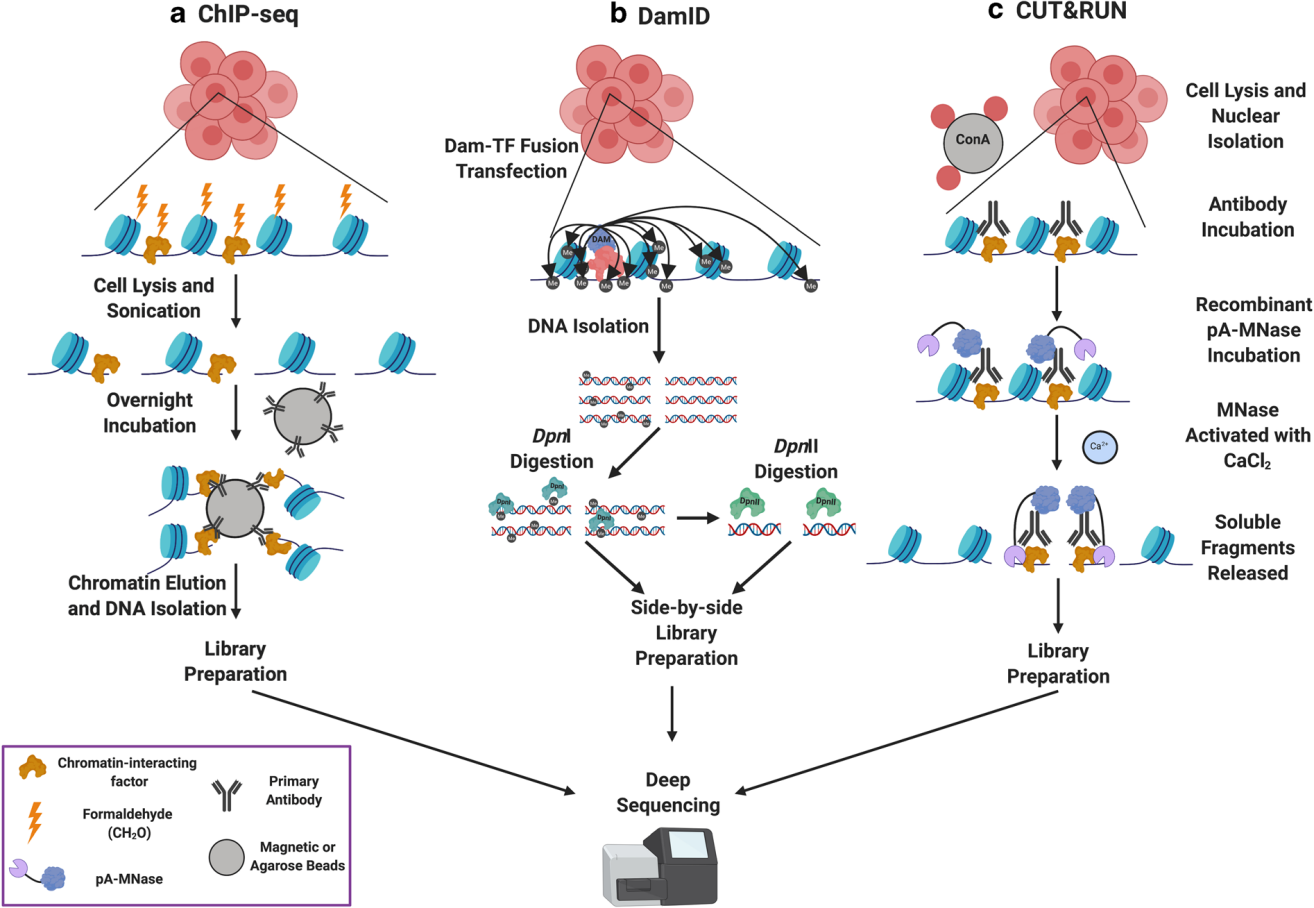
Methods for profiling protein localization on chromatin. **A** DamID exploits the *E. coli* DNA adenine methyltransferase (Dam) by fusing it to a factor of interest and transfecting that plasmid into a cell. This construct methylates adenines located near factor binding sites. Genomic DNA can then be isolated and digested with *Dpn*I, which specifically cleaves at the sequence G^m^ATC. A portion of the digested DNA is then digested with *Dpn*II, which cleaves unmethylated GATC to identify potential methylated sites out of Dam’s range. Side-by-side libraries are built and subjected to deep sequencing. **B** ChIP-seq is an antibody-based technology that begins with crosslinking of factors to DNA, followed by chromatin shearing and antibody pulldowns for the factor of interest on either magnetic or agarose beads. Crosslinks are then reversed, and DNA is isolated for deep sequencing. **C** CUT&RUN makes use of a recombinant Protein A-MNase (pA-MNase) fusion construct to bind to a primary antibody recognizing the factor of interest and specifically cleave DNA at factor binding sites, thereby creating small fragments that can be isolated from nuclei and used as a template for library construction and deep sequencing. CUT&RUN offers near-base pair resolution and can be carried out under native (i.e., non-crosslinking) conditions due to its high sequencing signal-to-noise ratio. Figure created with Biorender.com

Together, the chromatin profiling technologies that assess either accessibility or localization have been refined with increasing precision to improve target signal over background and to reduce necessary cell input in recent years, often reaching their peak with the development of single-cell adaptations of the techniques. Here, we review the technology development, methods, advantages and disadvantages, and optimization for low cell applications.

### Section 1: Methods in examining DNA accessibility and chromatin state

Eukaryotic DNA is compacted into the nucleus through interactions between DNA and histone proteins to form chromatin (Lammerding 2011). Generally, the basic repeating unit of chromatin, the nucleosome, poses a significant obstacle to DNA-templated processes, as factors are unable to occupy regions on DNA that are occluded by histone proteins (Beato and Eisfeld 1997; Felsenfeld 1992; Wallrath et al. 1994). Regions of open chromatin, however, are accessible to DNA-binding proteins and are often found at regulatory regions of the genome (Song and Crawford 2010; Thurman et al. 2012). Identifying regions of the genome that are accessible to non-histone proteins therefore provides important information for putative genomic regulatory regions, such as enhancers, promoters, and insulators as well as describing the nucleosome structure of known regulatory regions of the genome (Thurman et al. 2012).

**Fig. 3:**
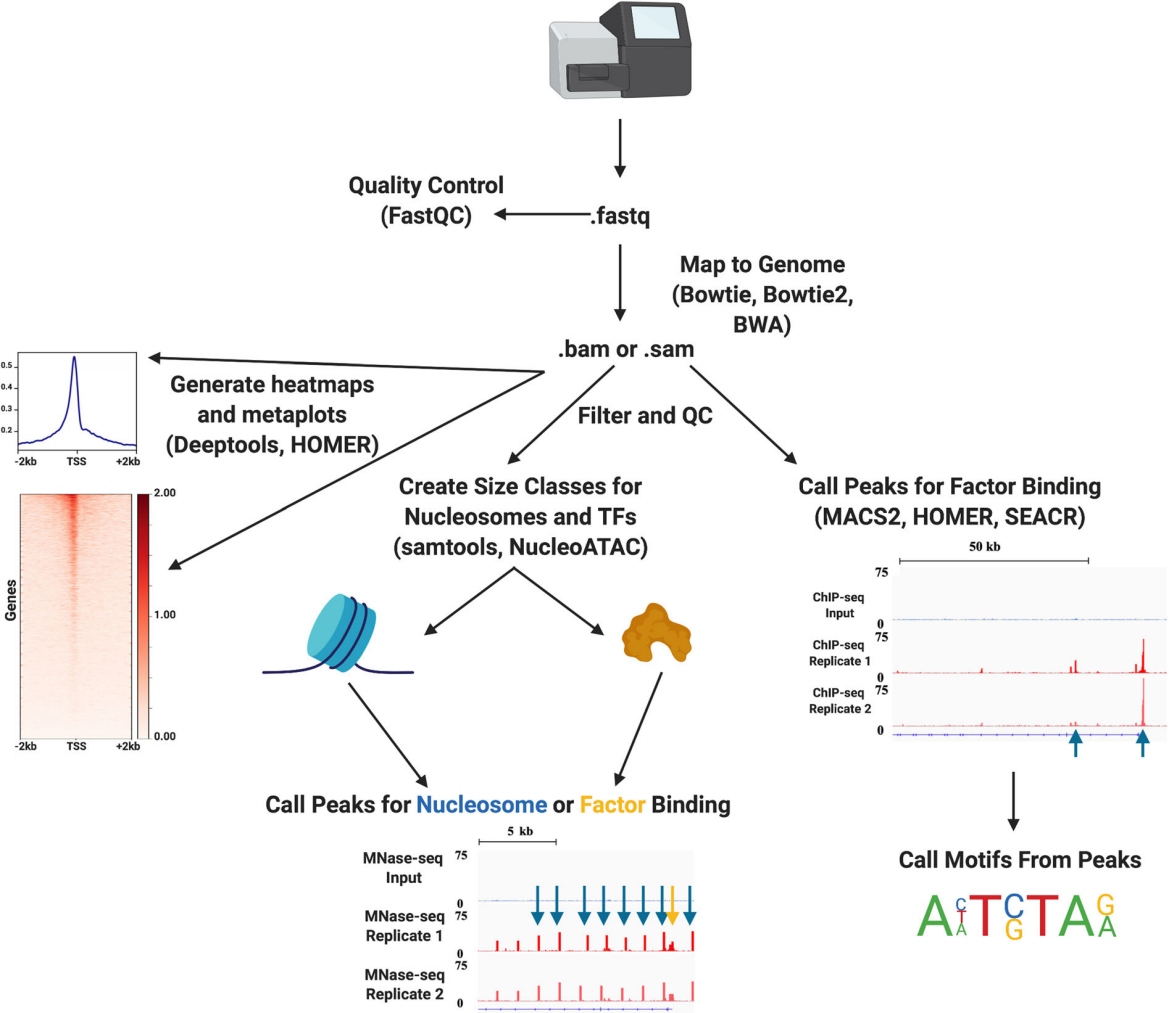
A general bioinformatic pipeline for analyzing genome-wide accessibility or profiling datasets. Although analyses vary depending on the technique used so as to minimize biases, we have presented a general pipeline for analyzing NGS-generated datasets. Following relevant quality control information (Andrews 2010), all sequencing experiments involve mapping to the genome of interest, generating files containing the sequence, alignment information, and quality information, known as .sam files (or, when compressed, .bam files; Langmead et al. 2009; Langmead and Saltzburg 2012; Li and Durbin 2009). These aligned files are filtered and used in downstream analyses; for studying nucleosome and factor occupancy and positioning, size classes are created to divide inaccessible regions by the factors blocking their availability (Li, Handsaker et al. 2009; Schep et al. 2015). From the size-divided accessibility .bam files and the quality-filtered localization .bam files, peaks can be called above local background scoring and/or compared with an input file (Heinz et al. 2010; Meers, Tenenbaum, and Henikoff, 2019; Zhang et al. 2008). From factor peaks, motifs can be called to determine which factors most likely bind these locations. Genomic data are typically viewed in the form of either heatmaps or metaplots (Heinz et al. 2010; Ramírez et al. 2016). Figure created with Biorender.com

Genomic methods used to examine chromatin accessibility have traditionally been based on preferential enzymatic digestion or modification of accessible DNA to DNA that is protected by bound histone proteins or transcription factors (Fig. 1). Many genomic accessibility techniques (e.g., DNase-seq and MNase-seq) have evolved from long-used nuclease footprinting experiments (Cappabianca et al. 1999; Dingwall et al. 1981; Galas and Schmitz 1978), taking advantage of next-generation sequencing developments to assess genome-wide nucleosome architecture rather than locus-specific footprinting (Crawford et al. 2006b; Schones et al. 2008). The techniques that have emerged are numerous, powerful, and capable of providing high-resolution data describing chromatin accessibility. For a general bioinformatic pipeline of how to asses these datasets, see Fig. 3. Though many of the enzymes used to profile accessibility bear slight biases, the portraits of genome architecture that emerge are generally consistent when compared with each other.

#### DNase-seq

DNase-seq is a method used to examine chromatin accessibility with the non-specific DNA endonuclease DNase I, which preferentially degrades DNA unprotected by bound proteins (e.g., histone proteins; Fig. 1A). Prior to DNase-seq, DNase I had been used for footprinting, in which a gel would be run after DNase treatment both in the presence and absence of the protein of interest; blank regions on the gel would be inferred to be protected and/or inaccessible regions, whereas more nucleosome-depleted—or accessible—regions would be marked by greater cleavage site presence on a gel (Cappabianca et al. 1999; Dingwall et al. 1981; Galas and Schmitz 1978). Francis Collins’ group first applied DNase I footprinting genome-wide in 2006, using microarray chips (DNase-chip) and massively parallel Sanger sequencing (Crawford et al. 2006a, b; Sabo et al. 2006). In 2008, Gregory Crawford’s group further developed this technology through combination with next-generation sequencing (Boyle et al. 2008) to greater success than the previous DNase-chip and DNase-seq experiments due to the increased resolution and quality offered over microarray technology. DNase-seq is applicable to all eukaryotic chromatin, including that of the common lab systems of plants, yeast, nematodes, flies, and mammalian cells.

DNase-seq is performed by isolating nuclei from cells, subjecting nuclei to general DNA digestion by DNase I, degrading RNA and proteins using RNases and Proteinase K, respectively, purifying the DNA using a phenol-chloroform extraction and ethanol precipitation, and gel-extracting fragments of sizes corresponding to the desired class of factors (typically 50–100 bp for transcription factors and 130–160 bp for nucleosomes; (He et al. 2014). Purified and size-selected DNA is then used as a template for library construction. Those regions least frequently identified in sequencing of DNase-seq libraries have been most frequently degraded by DNase I and are inferred to be most accessible.

There is an intrinsic bias for DNase I to degrade DNA differently based on sequence, and this effect has been suggested to be related to the width of the minor groove (Lazarovici et al. 2013). This limitation must be considered when preparing a DNase-seq experiment (He et al. 2014). For factors that are difficult to profile by DNase-seq, a recent modification has incorporated the use of 0.1% formaldehyde crosslinking to assist in identification, termed XL-DNase-seq (Oh et al. 2019). Another DNase-seq modification, single-cell DNase-seq (scDNase-seq) has applied DNase-seq to individual cells and low-input primary tissue samples (Jin et al. 2015). While similar to traditional DNase-seq, scDNase-seq has been further optimized, applying the following alterations: inclusion of bacterial carrier DNA, lack of nuclear isolation, optimized DNase I digestion, lack of agarose gel separation, and altered PCR conditions. These optimizations are designed to minimize sample loss and facilitate amplification of small DNA fragments (Cooper et al. 2017).

DNase-seq has been highly influential in identifying putative regulatory regions of the genome. Regions that seldom appear in DNase-seq libraries, known as DNase I hypersensitive sites (DHSs), are often used as a proxy for active regulatory regions, such as enhancers and promoters. Attempts to identify these DHSs have resulted in highly influential papers covering almost all known cis-regulatory regions, including over 2.9 million DHSs (Thurman et al. 2012) and over 45 million transcription factor occupancy events (Neph et al. 2012). Additionally, DNase-seq has become a valuable tool for investigating epigenetic tissue– and cell type–specific differences, largely through the efforts of the ENCODE project and the Roadmap Epigenomic Consortium (Consortium 2012; Maurano et al. 2015; Roadmap Epigenomics et al. 2015).

#### FAIRE-seq

As an alternative to DNase-seq to identify accessible regions throughout the genome, formaldehyde-assisted isolation of regulatory elements (FAIRE) was developed in 2007. Rather than digesting unprotected DNA, FAIRE relies on crosslinking of histones to DNA, while unbound DNA is inferred to be accessible (Fig. 1B). FAIRE was first developed for use with DNA microarrays (Giresi et al. 2007) but was soon combined with next-generation sequencing technologies (Gaulton et al. 2010). Similar to DNase-seq, FAIRE-seq can be used to examine regulatory regions (including TSSs, promoters, and enhancers), also referred to as DHSs. FAIRE-seq has been validated in plant, yeast, nematode, fly, mouse, and human cells.

A typical FAIRE-seq experiment involves formaldehyde crosslinking, with the most abundant crosslinking targets being histone proteins (Rodríguez-Gil et al. 2018; Simon et al. 2012). Crosslinked chromatin is then sheared by sonication to approximately 200–300 bp in size and DNA isolated via a phenol-chloroform extraction, wherein the highly crosslinked DNA remains in the organic phase and the non-crosslinked DNA is pulled to the aqueous phase. Non-crosslinked DNA from the aqueous phase can then be amplified and sequenced. Reads enriched in the sequencing pool tend to have lower nucleosome and factor binding and are therefore inferred to come from accessible regions.

A key disadvantage of FAIRE-seq experiments is that, while informative for histone-based chromatin architecture, regulatory regions that are bound by transcription factors or actively transcribed are also able to crosslink. The technique therefore relies on the presence of a mixed population for accurate accessibility profiling and is consequently lower resolution than the other techniques described in this review. As a result, fewer research groups have employed this technology; however, FAIRE-seq has been used to identify regulatory regions driving tumor development (Davie et al. 2015), to differentiate between ground-state and primed-pluripotent cells (Murtha et al. 2015), and, similarly, to the ENCODE and Roadmap Epigenomic Consortium’s DNase-seq efforts, to globally map accessible regulatory regions of chromatin (Bianco et al. 2015).

#### MNase-seq

MNase-seq is a method to assay nucleosome positioning and occupancy throughout the genome (Fig. 1C). Micrococcal nuclease (MNase) is an enzyme isolated from *Staphylococcus aureus* that displays both endo- and exonuclease activity to digest free DNA (Axel 1975; Dingwall et al. 1981). Similar to DNase I, MNase was used in DNA footprinting experiments to examine DNA accessibility before the invention of next-generation sequencing technologies (Cappabianca et al. 1999; Dingwall et al. 1981). MNase tiling arrays (MNase-chip) were used by Ollie Rando, Corey Nislow, and Frank Pugh’s groups, among others, to identify nucleosome positioning at high resolution before the advent of deep sequencing (Lee et al. 2007; Mavrich et al. 2008; Yuan et al. 2005). As with other techniques, MNase profiling was soon paired with next-generation sequencing technologies (Schones et al. 2008). MNase-seq has been used to map nucleosome architecture throughout eukaryotes from plants to yeast to humans.

An MNase-seq experiment begins with an in vivo formaldehyde crosslinking step that is designed to capture the interaction between proteins and DNA. This crosslinking allows bound proteins to shield their associated DNA from digestion by MNase. Following crosslinking, cells are lysed and digested with MNase, which is specifically activated by addition of Ca^2+^ to the lysis buffer. This digestion is halted by chelating the reaction, at which point the samples are RNase treated, crosslinks are reversed, and proteins are digested away from the chromatin. DNA is then isolated via a phenol-chloroform extraction and examined on an agarose gel to ensure proper digestion of the DNA without degradation. As the most abundant DNA-contacting proteins are histones, this gel will typically display periodic laddering every 147 base pairs, representing mono-, di-, and trinucleosomes, and so on.

Traditional MNase-seq protocols advise excision of the mono-nucleosome band to enrich for these protected DNA fragments (Cui and Zhao 2012b; Rando 2010; Zhang and Pugh 2011); however, it is also possible to perform deep sequencing on the entirety of a MNase-digested sample (Henikoff et al. 2011). Fragments remaining after MNase cleavage were protected from digestion and are therefore inferred to have been protein-bound. Sequencing DNA protected by all crosslinked proteins can provide additional footprinting corresponding to both small proteins (< 80 bp shielded from digestion, e.g., transcription factors) as well as the traditional nucleosome arrays (Hainer and Fazzio 2015; Henikoff et al. 2011).

Importantly, MNase displays different digestion kinetics based on the amount of enzyme used to digest a population of cells (Mieczkowski et al. 2016); in addition, in the case of some genomic loci (such as fragile nucleosomes), high and low digestion profiles can provide drastically different information (Chereji et al. 2017; Mieczkowski et al. 2016; Weiner et al. 2010). It is therefore crucial to perform MNase-seq experiments on a uniform population with no-MNase, low-MNase, and high-MNase replicates. While MNase-seq has traditionally been limited by cellular input available, single-cell MNase-seq has recently been published (Lai et al. 2018).

MNase has a well-documented preference for cleavage of AT-rich naked DNA (Chung et al. 2010); however, this sequence preference is minute compared with preference due to chromatin accessibility (Allan et al. 2012). Nonetheless, techniques are available that can minimize bias due to MNase preference. Jay Shendure’s lab has published an alternative, single-stranded library building protocol for MNase-seq, known as MNase-SSP that displays low sequence bias and enriches for shorter fragments than traditional MNase-seq, making for robust profiling of transcription factors (Ramani et al. 2019). In addition, a few closely related alternatives have been developed that utilize chemical cleavage of DNA, rather than enzymatic digestion. MPE-seq, developed by Bing Ren’s group, uses methidiumpropyl-EDTA-Fe(II) (MPE) to preferentially cleave linker DNA between histones (Ishii et al. 2015). Steve Henikoff’s group has also developed a chemical DNA cleavage technique, using a mutation in H4 (S47C) to create a site-specific nuclease by phenanthroline-mediated chelation of copper, which locally cleaves DNA at the dyad axis in the presence of peroxide (Chereji et al. 2018).

MNase-seq has been used to profile nucleosome occupancy and positioning changes at regulatory regions as a result of cellular differentiation, highlighting key changes in embryonic stem cell enhancers (West et al. 2014). Furthermore, MNase-seq can even be used to profile paused Pol II positioning, a trend that has been confirmed by parallel Pol II ChIP-seq (Teves and Henikoff 2011). Interestingly, MNase-seq profiling can be used to reliably predict 3D genome interactions and higher-order chromatin structures (Schwartz et al. 2019; Zhang et al. 2017). Because of its ability to capture transitory interactions via crosslinking, MNase-seq is one of the most versatile chromatin accessibility profiling techniques.

#### ATAC-seq

The assay for transposase accessibility and deep sequencing (ATAC-seq) is an additional technology to assess accessible chromatin. ATAC-seq involves the use of a hyperactive Tn5 transposase to insert sequencing adapters into open regions of chromatin to then sequence those regions through next generation sequencing (Buenrostro et al. 2013) Fig. 1D). Unlike other accessibility-profiling techniques, ATAC-seq was only recently developed (Buenrostro et al. 2013), though it has been adapted for use at a single locus (ATAC-qPCR; (Yost et al. 2018). Although ATAC-seq is a relatively new technique, the enzyme used, Tn5 transposase, was one of the first transposases identified, and has been used for in vitro transposition experiments for over 20 years (Goryshin and Reznikoff 1998; Naumann and Reznikoff 2002; Reznikoff 2003; Reznikoff 2008). Tn5 operates by a DNA-mediated “cut-and-paste” mechanism, wherein the transposase excises a segment of DNA, binds to a target DNA site, induces a double-strand break, and inserts the transposon into the new locus (Ivics et al. 2009). In ATAC-seq, Tn5 is loaded with a transposon designed to add sequencing adapters at the insertion point, forming a functional transposome. ATAC-seq has been used to map open chromatin in yeast, plants, nematodes, flies, mammals, and even frozen tissues (Corces et al. 2017).

ATAC-seq is performed in two to three basic steps consisting of cellular lysis and DNA transposition steps and DNA extraction and amplification (Buenrostro et al. 2013). Various ATAC-seq protocols have been developed including the original ATAC-seq (Buenrostro et al. 2013), FAST-ATAC-seq, which was designed for blood cells (Corces et al. 2016), and Omni-ATAC-seq (Corces et al. 2017), largely differing in the detergents used in cellular lysis. Because ATAC-seq relies on insertion to accessible DNA, rather than digestion of protected DNA, the technique is prone to sequencing contamination by mitochondrial DNA. Because of this prevalence, methods have been developed to reduce mitochondrial reads in ATAC-seq (Corces et al. 2017; Montefiori et al. 2017; Rickner et al. 2019).

ATAC-seq has successfully been used to assess chromatin accessibility in single cells (Buenrostro et al. 2015; Mulqueen et al. 2019) and from frozen tissue (Corces et al. 2017), and therefore the technique is be a valuable tool for confronting core genomic issues of cell heterogeneity and low sample availability. Indeed, Jay Shendure’s group has published 85 different chromatin accessibility patterns (largely cell type-specific) based on single-cell indexed ATAC-seq in various mouse tissues (Cusanovich et al. 2018). In addition, Howard Chang’s and William Greenleaf’s groups have published accessibility studies in a litany of primary human cancers using ATAC-seq (Corces et al. 2018). ATAC has further been paired with visualization and flow cytometry (ATAC-see) to allow direct imaging, quantitation, and cell sorting as results of genome accessibility (Chen et al. 2016).

#### Summary

Techniques used to measure chromatin accessibility rely on two basic principles: first, that proteins can shield DNA from digestion and second, that histone proteins are the most prominent proteins interacting with DNA. DNase-seq, MNase-seq, and ATAC-seq fundamentally rely on the first principle, while FAIRE-seq and MNase-seq rely more on the second principle; however, both principles are important to the discrete patterns of accessibility uncovered by each technique. The aforementioned techniques provide distinct—yet consistent—snapshots of nucleosome positioning and chromatin accessibility, and each technique has particular advantages and disadvantages (Table 1). These technologies have illuminated and verified the accessible state of the genome by orthogonal approaches and led to identification of approximately 3 million putative regulatory regions of the human genome (Thurman et al. 2012).

**Table 1:** Considerations when choosing a genome accessibility or profiling technique. Although many of the techniques described in this review have been optimized for single-cell input, typical cellular input tends to be much higher. A few advantages and disadvantages for each technique have been listed, as well as references for papers that have been highly influential in the method’s development and refinement

Technique	Typical cell input	Minimal cell input	Approximate sequencing coverage necessary for mammalian genome	Genomic target	Advantages	Disadvantages	References
DNase-seq	≥ 1 M cells	1 cell	20–50 M reads	Open chromatin	DHSs are the gold standard for identification of regulatory regions	High cell input typically required	(Cooper et al. 2017; Crawford et al. 2006b)
FAIRE-seq	≥ 100,000 cells	100,000 cells	20–50 M reads	Nucleosome occupancy	Fast and easy protocol	Low signal-to-noise ratio Highly dependent on correct crosslinking efficiency	(Giresi et al. 2007; Tsompana and Buck 2014)
MNase-seq	≥ 1 M cells	1 cell	40–60 M reads	Nucleosome and TF occupancy and positioning	TF and nucleosome binding information	Indirect detection of active regulatory regions High cell input typically required	(Lai et al. 2018; Mueller et al. 2017 Schones et al. 2008)
ATAC-seq	≥ 50,000 cells	1 cell	40–60 M reads	Open chromatin	Fast protocol Native conditions	Requires high sequencing coverage to accurately map factors High prevalence of mitochondrial read contaminants	(Buenrostro et al., 2013, 2015; Corces et al. 2017)
ChIP-seq	≥ 500,000 cells	100–10,000 cells	20–40 M reads	Protein localization	Most common profiling technique Numerous protocols and comparative datasets available	Mapping resolution limited by chromatin shearing efficiency Limited by quality of antibody	(Albert et al. 2007; Cao et al. 2015)
DamID	≥ 10,000 cells	1 cell	10–40 M reads	TF localization 3D genome contacts	Not antibody-dependent	Dependent on GATC presence Does not profile endogenous protein Low base-pair resolution because of extensive Dam range of action	(Kind et al. 2015; van Steensel and Henikoff 2000)
CUT&RUN	≥ 100,000 cells	1 cell	10 M reads	Protein localization	High signal to noise ratio Low cellular input necessary Native conditions	Limited by quality of antibody	(Hainer et al. 2019; Skene and Henikoff 2017)

In parallel to mapping generally accessible regions of the genome, investigating the factors that interact with chromatin and regulate these accessible regions through factor-specific protein localization profiling is equally important to understanding the basic principles of genome architecture.

### Section 2: Methods in protein localization profiling on chromatin

Depending on their specific roles within the nucleus, chromatin-interacting proteins display characteristic patterns of genomic localization. By identifying the genomic regions at which proteins are found, it is possible to identify functional roles, motifs important for binding, and regulatory networks of DNA-templated processes in vivo. Like methods of measuring DNA accessibility, there are numerous approaches to identifying genomic binding sites of chromatin-interacting proteins that have gained popularity in recent years (Fig. 2), each of which has advantages and disadvantages (Table 1). Broadly, profiling methods must balance resolution of binding site identification with sample necessary to perform the experiment. Some methods, like ChIP-exo (Rhee and Pugh 2012), prioritize base-pair resolution, at the expense of increased necessary sample input; others, like DamID (van Steensel and Henikoff 2000), provide robust interaction data without the input limitations of higher-resolution techniques. More recently, techniques derived from the chromatin immunocleavage (ChIC) method (Schmid et al. 2004) have emerged and are capable of providing high-resolution identification of binding sites with even ultra-low input samples. For a general bioinformatic pipeline on how to identify these genomic binding sites, see Fig. 3.

#### ChIP-seq

The most commonly used technique to assess the localization of chromatin-binding proteins, chromatin immunoprecipitation (ChIP) (Fig. 2A), was developed for use at a single locus using radioactive DNA labeling by Gilmour and Lis (1984) and formaldehyde crosslinking and gel-based imaging by Solomon and Varshavsky (1985). This technique had been in use for many years before being adapted for deep sequencing after library construction to examine genomic identification of a chromatin-interacting protein’s binding site (Albert et al. 2007). Based on the initial radiolabeling experiments, ChIP-chip, a technique in which ChIP DNA is hybridized to DNA microarrays against various genomic loci, was developed in 2000 as the first broad genomic application of ChIP (Ren et al. 2000). ChIP was combined with quantitative PCR (ChIP-qPCR) as a way to examine protein occupancy at multiple locations in a quantitative manner that was more targeted than ChIP-chip, but less restrictive than single-locus radiolabeled ChIP (Irvine et al. 2002). ChIP-seq robustly profiles protein-DNA interactions throughout eukaryotic species.

A ChIP experiment typically begins with a formaldehyde incubation designed to crosslink the lysines of interacting proteins with local DNA. Cells are then lysed to release crosslinked chromatin and subjected to unbiased sonication to shear the chromatin into short segments (typically between 100 and 400 base pairs). The sheared chromatin is then incubated with an antibody targeting the protein of interest followed by addition of a secondary IgG recognizing antibody that is typically coupled to sepharose or magnetic beads. Upon recognition of the epitope, the interacting region of DNA is pulled down with the protein to which it is crosslinked, thereby specifically isolating regions of DNA at which the protein crosslinks (and to which the protein is necessarily in close proximity—approximately 2 Å; (Perez-Romero and Imperiale 2007). Crosslinks are then reversed, protein is digested, and the DNA is isolated to be used as a template for locus-specific qPCR or to be run on a gel.

ChIP-seq has been combined with various techniques to provide heightened resolution, including lambda exonuclease digestion (ChIP-exo and ChIP-nexus; (He et al. 2015; Rhee and Pugh 2012), UV-crosslinking (UV-ChIP; (Gilmour et al. 1991), and MNase digestion (Native ChIP; (O’Neill 2003). ChIP-exo and ChIP-nexus are two techniques that utilize nuclease digestion to improve ChIP-seq resolution to a near-base-pair level. ChIP-exo uses lambda exonuclease to digest unbound dsDNA 5′-3′ until reaching a protein-DNA crosslink through which the nuclease cannot proceed (Rhee and Pugh 2012). Similar to ChIP-exo, ChIP-nexus relies on digestion of crosslinked DNA using lambda exonuclease; however, ChIP-nexus also incorporates a modified library build protocol and a barcode-based monitor of overamplification (He et al. 2015). In addition, ChIP-nexus requires only one 3′ sequencing adaptor, reducing input requirements relative to traditional ChIP-seq (He et al. 2015). UV-ChIP utilizes UV light as a zero-length in vivo crosslinking agent that tests direct protein interaction; however, UV crosslinking provides low yields, making it unsuitable for low-input samples or infrequent interactions (Toth and Biggin 2000). Native ChIP uses MNase digestion as a gentler alternative to sonication that allows for identification of protein binding on non-crosslinked chromatin, and at substantially higher resolution than traditional ChIP-seq because it is no longer limited by sonication efficiency (O’Neill 2003).

The most pressing limitation to ChIP-seq experimentation is input; to produce a high signal-to-noise ratio, ChIP-seq typically requires millions of input cells, particularly to examine transcription factor binding. As histones are far more abundant than other DNA-binding proteins, optimizing ChIP-seq technologies for low input has been far more fruitful using histones than factors. For traditional, crosslinking-based ChIP-seq techniques, μChIP-seq has been sufficient to profile histone modifications in 400 cells (Dahl et al. 2016), although ChIP has been paired with microfluidics technology (Cao et al. 2015; Rotem et al. 2015) to reduce necessary input to 100 cells for profiling histone modifications. Native ChIP-seq techniques have been more successful in reducing cellular input due to gentler chromatin shearing. In 2006, Carrier ChIP was successfully used to profile histone modifications in 50 cells (albeit with millions of “carrier” cells to reduce sample loss; (O’Neill et al. 2006), while more recent attempts have reduced cellular input for histone modification profiling to 500 cells (MINT-ChIP and ULI-NChIP) and 200 cells (STAR-ChIP; (Liu et al. 2016; van Galen et al. 2016; Zhang et al. 2016). While transcription factors’ abundance and transitory binding make them harder to profile in low-input samples, two ChIP-based techniques have been successfully lowered cell input: ChIPmentation and Carrier-assisted ChIP-seq. The first, ChIPmentation, was developed by Christoph Bock’s group and utilizes Tn5 transposase to ligate sequencing adapters directly onto chromatin on beads (Schmidl et al. 2015); ChIPmentation was used to profile transcription factors in 100,000 cells. In addition, Jason Carroll’s group has used carrier-assisted ChIP-seq to profile transcription factor localization in as few as 10,000 cells (Zwart et al. 2013).

As one of the first and most prominent genomic techniques, ChIP and its derivatives have been extraordinarily impactful in understanding regulation of chromatin interactions and transcription. To date, the term “chromatin immunoprecipitation” has almost 23,000 PubMed hits and over 9000 publicly available datasets in the ENCODE database, with far more stored in the NCBI Sequence Read Archive (Consortium 2012). Although ChIP-seq remains the gold standard of factor localization profiling, other techniques have been developed over the past 30 years to examine factor localization through different approaches.

#### DamID

DamID presents a non-ChIP alternative to locating proteins on chromatin (Fig. 2B) (van Steensel and Henikoff 2000). DamID makes use of a recombinant protein (*Escherichia coli* DNA Adenine Methyltransferase or Dam) fused to the chromatin-interacting protein of interest to identify genomic regions at which the protein interacts. Dam methylates adenine within the sequence GATC (Barras and Marinus 1989; Boivin and Dura 1998; Wines et al. 1996). As adenine methylation does not occur in most eukaryotes, DamID provides a native and specific readout for factor localization (Barras and Marinus 1989). Dam methylation can spread up to 5 kb from the protein-binding site (van Steensel and Henikoff 2000), highlighting the tradeoff between resolution and specificity balanced in DamID experiments. Additionally, more accessible regions of the genome are more likely to be methylated by Dam (Greil et al. 2006), a variable that is controlled for by profiling with transfection of unfused Dam. Although DamID was pioneered with Southern blotting and quantitative PCR (qPCR) as methylation quantitation, they have since been supplanted by next-generation sequencing technologies (Aughey et al. 2019; Greil et al. 2006). DamID is most commonly applied in *Drosophila* cells but has been used in yeast, *C. elegans*, *Arabidopsis*, mice, and human cells, illustrating a more versatile range of profiling.

A typical DamID experiment involves construction of a plasmid with Dam fused to the N- or C-terminus of the protein of interest. The plasmid is then transfected into the cells to be examined, as are a control plasmid containing Dam alone and an empty vector. Genomic DNA is then isolated from the transfected cells and digested with the *Dpn*I restriction enzyme. As *Dpn*I exclusively and specifically digests G^m^ATC, fragments generated from this digestion are inferred to have been in close proximity to the chromatin-interacting protein of interest. Adapters are ligated to the *Dpn*I-digested fragments, and the DNA is then treated with *Dpn*II, a restriction enzyme that cleaves only unmethylated GATC, to doubly select for G^m^ATC in the genome. DNA libraries are then amplified and can be submitted for deep sequencing.

DamID has not reached the same popularity as ChIP-seq but presents some notable strengths. First, DamID is not dependent on antibodies to profile factor binding, a significant advantage for profiling understudied proteins. Additionally, DamID was the first method by which one could confirm ChIP data by an alternate approach. DamID is, however, disadvantaged by the fact that the profiled protein is not endogenous to the host cells. The binding sites of a Dam fusion construct will often be comparable with an endogenous protein, but likely not identical due to the presence of the Dam construct itself as well as its plasmid-based expression. Additionally, DamID requires a genetically tractable system that can be transfected with the Dam fusion plasmid. Furthermore, DamID is limited by its low resolution; because Dam can methylate residues up to 5 kb from the fusion protein’s binding site, and extensive false positives can be found (van Steensel and Henikoff 2000). Because of this range of methylation, DamID is unlikely to reach the resolution offered by ChIP-based techniques; DamID is not, however, constrained by the same input limitations, and has been used to profile transcription factor binding from 1000 ES cells (Tosti et al. 2018) and even single cells (Lai et al. 2019). Although ChIP-seq (and more recently, CUT&RUN) has largely superseded DamID for factor localization, DamID is becoming more popular in studying broader chromatin features; for instance, Chromatin Accessibility Targeted DamID (CATaDA) has been developed to assess open chromatin (Aughey et al. 2018). CATaDa utilizes an untethered Dam protein to methylate regions of open chromatin, leaving nucleosome-bound DNA unmethylated (Aughey et al. 2018). Split DamID has also been used to profile co-occupancy of two proteins at genomic loci, acting in a similar manner to a yeast two-hybrid screen (Hass et al. 2015), and a catalytically inactive *Dpn*I-GFP fusion construct has been used to examine Dam-driven GATC methylation in real-time using microscopy (Kind et al. 2015).

#### CUT&RUN

Cleavage under targets and release using nuclease (CUT&RUN) was developed by Skene and Henikoff in 2017 as a genome-wide modification of Ulrich Laemmli’s group’s 2004 ChIC technique, in which a recombinant Protein A fused to micrococcal nuclease (pA-MNase) can be combined with a primary antibody to specifically target MNase and cleave DNA surrounding sites where the protein of interest binds (Fig. 2C; (Schmid et al. 2004). Similar techniques include chromatin endogenous cleavage (ChEC; (Schmid et al. 2004), in which involves a C-terminal fusion of MNase to a protein of interest and ChEC-seq, a genome-wide pairing of ChEC and next-generation sequencing (Zentner et al. 2015). While ChEC has been successfully applied to assess the localization of multiple proteins (Baptista et al. 2017; Grunberg et al. 2016; Grunberg and Zentner 2017; Warfield et al. 2017; Zentner et al. 2015), the technique is limited by a need to specifically tag the protein of interest. CUT&RUN, on the other hand, utilizes a recombinant pA-MNase protein to recognize any primary antibody with compatible IgG backbones. Although CUT&RUN is a recently developed technique, it has been used to profile protein-DNA interactions in *Arabidopsis*, yeast, flies, mice, and human cells, demonstrating a versatile range of application.

A CUT&RUN experiment involves either a nuclear isolation with a hypotonic buffer to lyse the cells (Hainer and Fazzio 2019; Skene and Henikoff 2017) or cell permeabilization with digitonin (Skene et al. 2018) and lectin-coated concanavalin A magnetic beads to isolate the nuclei. Subsequent steps are carried out in the bead-bound nuclei until the protected DNA fragments are released prior to library preparation. Primary antibody targeting the protein of interest is added and allowed to freely diffuse into the nuclei, followed by addition of recombinant pA-MNase, which recognizes the IgG backbone of the primary antibody and is therefore specifically directed to the protein of interest’s binding sites on chromatin. The MNase is then activated by addition of Ca^2+^ and digested in an ice-water bath (for sub-optimal MNase digestion kinetics) to cleave DNA and release the protein-bound fragments into the supernatant. Released fragments are then RNase treated, digested with Proteinase K, purified, and used as input for library construction. CUT&RUN experiments are performed in tandem with a replicate in which the primary antibody is either left out of the sample or replaced with an IgG control, measuring background cutting by the free pA-MNase construct and correcting for an inherent bias towards more accessible regions of the genome. In addition, heterologous DNA can be spiked-in to the reaction upon chelating the MNase digestion (Skene and Henikoff 2017) or contaminating *E. coli* DNA from the pA-MNase purification can be used as a spike in (Meers et al. 2019). CUT&RUN provides a high signal-to-noise ratio, with the reduced background allowing thorough sequencing with approximately 10 million reads, whereas a ChIP-seq experiment requires 20–40 million reads to accurately assess protein binding.

CUT&RUN has proven to be adaptable to numerous alterations to suit experimental contexts, most of which have been developed by Steve Henikoff’s group. One such adaptation is robotic automation of the protocol for high-throughput profiling (AutoCUT&RUN; (Janssens et al. 2018). In addition, Henikoff’s group has published CUT&RUN.Salt, a method that allows chromatin fractionation based on solubility and is especially useful for profiling centromeric or otherwise insoluble chromatin under typical conditions (Thakur and Henikoff 2018). To improve efficiency of pA-MNase-antibody binding, Henikoff’s group has engineered a recombinant Protein A-Protein G-MNase fusion construct that allows for profiling of non-rabbit antibodies without a secondary antibody step (Meers et al. 2019). Finally, CUT&RUN has been combined with traditional ChIP (CUT&RUN.ChIP) that allows one to ChIP for protein complexes present within released CUT&RUN fragments (Brahma and Henikoff 2019). The general CUT&RUN technique therefore appears flexible to profile protein localization for a variety of experimental designs and desired outcomes.

In 2019, the first single-cell genome-wide profiling of chromatin-bound proteins using CUT&RUN was published to examine pluripotency factors in murine embryonic stem cells (Hainer et al. 2019). In addition to profiling in single cells, factor binding was profiled in individual early blastocysts (consisting of between 30-50 cells each), an application not previously possible using ChIP-based techniques. More recently, Cleavage Under Targets and Tagmentation, or CUT&Tag, was developed as a modification on CUT&RUN that uses a recombinant Protein A-Tn5 transposase fusion instead of a recombinant pA-MNase fusion protein (Kaya-Okur et al. 2019). CUT&Tag has been used to profile histone modifications in single cells, although it has not yet been used to profile transcription factor binding in single cells (Kaya-Okur et al. 2019). In addition to CUT&Tag, a similar single-cell modification of ChIC, scChIC-seq, which involves tethering of MNase to a specific antibody and cleavage of target sites using the antibody to direct the MNase, then selectively amplifying cleaved fragments by PCR was developed (Ku et al. 2019). Between CUT&RUN, uliCUT&RUN, CUT&Tag, ChEC-seq, and ChIC-seq, ChIC- and ChEC-derived techniques appear poised to facilitate the next era of chromatin-interacting factor profiling.

#### Summary

As genomic technique refinement has allowed researchers to identify factor binding sites on chromatin and DNA accessibility with high resolution, the limitations of standard techniques have become more and more apparent. Because of differences due to cellular heterogeneity, inconsistent enzyme digestion kinetics, and untargeted sample isolation, recent advances in genomic techniques have focused on reducing necessary sample input and background signal. These technical improvements have made it possible to examine genome architecture and factor-binding profiles in individual cells, low-input samples like patient biopsies, and subsets of heterogeneous cellular populations. What has emerged from genomic studies of accessibility and factor binding is a complex picture of DNA templated activities regulated by chromatin architecture.

Profiling of genome accessibility and factor binding has set the stage for identification of genomic regulatory mechanisms; however, these techniques are merely a start towards understanding the gene regulation on a mechanistic level. These data must be integrated to understand how transcriptional and cellular networks function cooperatively and antagonistically to shape the functional genome. Additionally, comparisons between cell types will be important to provide insight into the ways in which a common suite of factors drive cell type-specific functions.

## Abbreviations

DHS: DNase I hypersensitive site
DNase-seq: DNase I coupled with deep sequencing
XL-DNase-seq: Crosslinking DNase I coupled with deep sequencing
scDNase-seq: Single-cell DNase I coupled with deep sequencing
FAIRE-seq: Formaldehyde-assisted isolation of regulatory elements
MNase-seq: Micrococcal nuclease digestion coupled with deep sequencing
MPE-seq: Methidiumpropyl-EDTA cleavage coupled with deep sequencing
ATAC-seq: An assay for transposase accessibility
ChIP-seq: Chromatin immunoprecipitation coupled with deep sequencing
ChIP-exo: Chromatin immunoprecipitation coupled with lambda exonuclease digestion
μChIP: Micro-ChIP
STAR-ChIP: Small-scale TELP-assisted rapid chromatin immunoprecipitation
MINT-ChIP: Multiplexed, indexed T7 chromatin immunoprecipitation
ULI-ChIP: Ultra-low input ChIP
DamID: DNA adenine methyltransferase identification
ChIC: Chromatin immunocleavage
ChEC: Chromatin endogenous cleavage
CUT&RUN: Cleavage under targets and release using nuclease
ENCODE: Encyclopedia of DNA elements
